# To study the mechanism of panax notoginseng in the treatment of aspirin resistance in the secondary prevention of stroke based on TLR4/MyD88/NF-κB signaling pathway: A study protocol

**DOI:** 10.1097/MD.0000000000031919

**Published:** 2022-12-16

**Authors:** Hui Wang, Jie Yuan, Ying Wang, Jie Chen

**Affiliations:** a Tianjin University of Traditional Chinese Medicine, Tianjin, China; b Geriatric Department, Xi’an Hospital of Traditional Chinese Medicine (Xi’an Affiliated Hospital of Shaanxi Provincial Hospital of Chinese Medicine), Xi’an, China; c Shanxi Provincial Hospital of Chinese Medicine (Shaanxi Academy of Traditional Chinese Medicine), Xian, China; d Department of Encephalopathy, Shaanxi Hospital Province of Traditional Chinese Medicine, Xian, China; e School of Basic Medical Sciences, Chengdu University of Traditional Chinese Medicine, Chengdu, China; f Shaanxi University of Traditional Chinese Medicine, Shaanxi Province, Xianyang, China.

**Keywords:** aspirin resistance, Panax notoginseng (Sanqi), platelet aggregation rate, RCT, stroke, study protocol, TLR4/MyD88/NF-κB

## Abstract

Aspirin, as an typical antiplatelet therapy for secondary stroke prevention, have been proved that can significantly reduce incidence and recurrence of cerebrovascular ischemic events. However, due to drugs biological characteristics, aspirin resistance (AR) often occurs in clinical practice, which significantly influence secondary prevention in stroke patients. The growing evidence of activating blood and removing stasis herbs medicine *(Sanqi*) for AR is promising. However, the efficacy and mechanism of *Panax notoginseng (Sanqi*) for AR in secondary stroke prevention has not been confirmed.

**Methods/design:**

This is a prospective 2-center, assessor and statistician blinded, randomized, controlled trial. We will allocate 106 subjects aged between 45 and 65 years old, diagnosed with aspirin semi-resistance after stroke to 2 groups randomly in a ratio of 1:1. Patients in the experimental group will be treated with conventional treatments plus *Panax notoginseng (Sanqi*) while the others in the control group will be treated with only conventional treatments. All will be given different medications for 30 days. Patients will be measured with the platelet aggregation rate and serum TLR4, MyD88, NF-*κ*B, COX-2, IL-6, CRP, TXB2 level for clinical efficacy and mechanisms at baseline and the 14^th^, 30^th^ day of treatment. Baseline characteristics of patients will be summarized by groups and compared with Chi-square for categorical variables, and Student’s independent *t* test or nonparametric Mann-Whitney *U* test for the continuous variables. Primary and secondary outcomes will be analyzed with 2-way repeated measures Anova, and Post Hoc test.

**Conclusion:**

The present study aims to investigate short-term add-on efficacy and mechanism of *Panax notoginseng (Sanqi*) for aspirin resistance in secondary stroke prevention via TLR4/MyD88/NF-κB signaling pathway. With this, we expect to find out an appropriate partial substitute of aspirin for aspirin resistance individuals.

**Trial Registration:**

The trial was registered on Chinese Clinical Trial Registry (http://www.chictr.org.cn/index.aspx) with the ID ChiCTR2100045773 at April 24, 2021.

## 1. Introduction

Despite the rapid development of medicine, stroke is still a major cause of death worldwide.^[1]^ Platelet activation still plays an important physio-pathological role in cerebrovascular thromboembolism events. Therefore, antiplatelet drugs, especially aspirin, play a vital role in the secondary stroke prevention.^[2]^ Since the 1990s, it has been found that about 5% to 60% patients still exist stroke recurrence when taking aspirin orally in clinical practice. This phenomenon has been called “aspirin resistance (AR)^.^”^[3]^ Currently, AR is divided into clinical AR and laboratory AR. Clinical AR refers to that it failures to inhibit thromboxane A2 (TXA2) production and TXA2- dependent platelet aggregation after regular aspirin administration. Laboratory AR is defined as arachidonic acid -induced mean platelet aggregation rate ≥ 20% and adenosine diphosphate -induced mean platelet aggregation rate ≥ 70%. Those who meet 1 of above 2 criteria are aspirin semi-resistance (ASR) ones.^[4]^ It has been reported that AR may increase the risk of recurrent cerebrovascular events by 2 times and the risk of death by 3.5 times,^[5]^ which brings heavy economic burden to the society and families.

Mechanisms of AR are multiple and complex, including clinical, genetic, pharmaco- logical, and biological factors.^[4]^ How to enhance bioavailability to overcome AR, there is no unified standards. It may mainly increase dose and frequency of drug, or combine with other antiplatelet drugs, which are lack of effective medical evidence,^[6]^ and increase risk of hemorrhage. Therefore, it’s urgent to find a safe and effective partial substitute of aspirin for AR individuals.

Studies have shown that some activating blood and removing stasis herbs medicine have a good prospect for AR.^[7]^ Long-term clinical observation, *Panax notoginseng (Sanqi*) can improve platelet aggregation rate of stroke patients with AR. However, whether it is effective for AR patients and how it works need to be further studied. Based on the current cases analysis, aspirin combined with *Panax notoginseng (Sanqi*) powder, which reduce platelet aggregation rate, serum TXB2 level and increase PGI2 level, is superior to aspirin alone among AR patients. Besides, literatures about the relationship between inflammation and TLR4/MyD88/NF-*κ*B signaling pathway of activating blood and removing stasis herbs medicine in AR are a growing.^[8]^ Moreover, *Panax notoginseng (Sanqi) shows* the anti-inflammatory and antiplatelet effect in experimental study.^[9]^ Thus, we proposes that *Panax notoginseng (Sanqi*) may reduce IL-6, COX-2 and TXA2 level to improve AR by regulating the inflammatory TLR4/MyD88/NF-*κ*B signaling pathway, so as to clarify the specific mechanism of *Panax notoginseng (Sanqi*) for AR and provide a new clinical molecular target for AR.

The primary objective of this study aims to investigate short-term add-on efficacy and mechanism of *Panax notoginseng (Sanqi*) for aspirin resistance in secondary stroke prevention via TLR4/MyD88/NF-*κ*B signaling pathway with a more strictly randomized controlled trial (RCT).

## 2. Methods/design

### 2.1. Study design

This is a prospective 2-center, assessor and statistician blinded, randomized, controlled trial with a parallel 2-arm group from June 2021 to May 2023. This study protocol is draw up based on Recommendations for Interventional Trials (SPIRIT) 2013 statement.^[10]^ The SPIRIT checklist will be presented in (Table S1, Supplemental Digital Content, http://links.lww.com/MD/H991). A total of 106 participants will be randomized into 2 groups at a ratio of 1:1. Participants in the treatment group will be treated with conventional treatments plus *Panax notoginseng (Sanqi*). Participants in the control group will be treated with conventional treatments.

**Figure 1.**
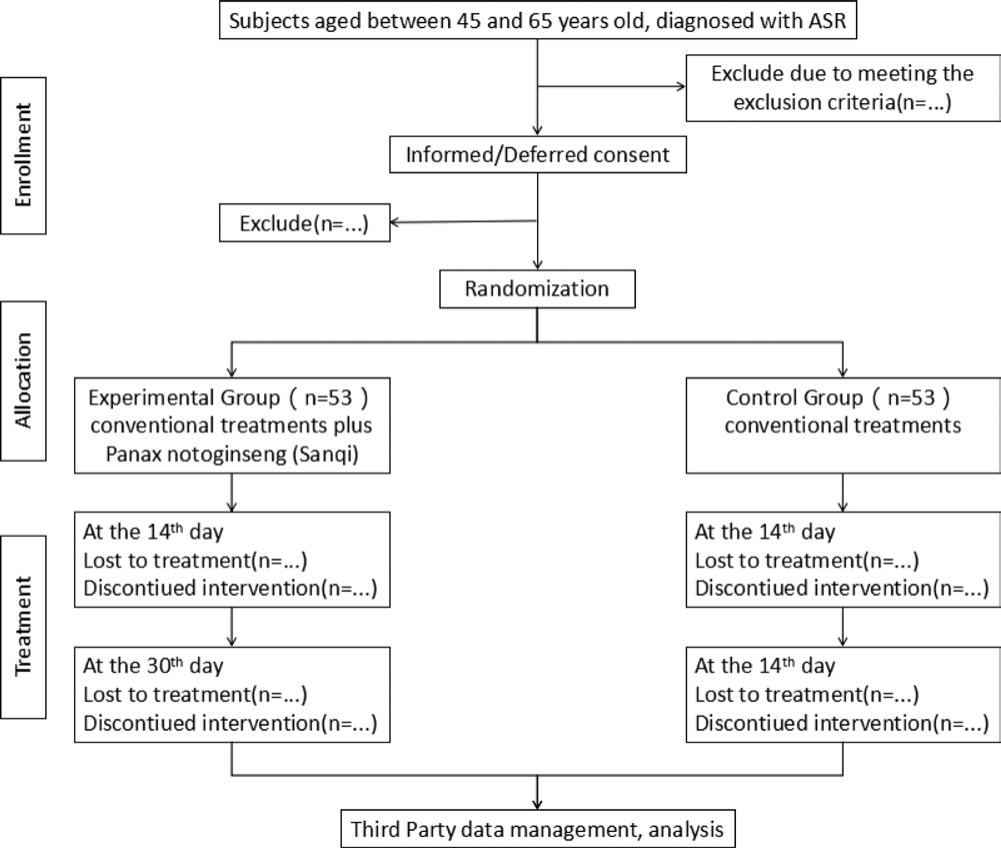
Participant flowchart.

The whole study period is 35 days, including 5-days baseline observation, 30-days treatment. Subjects will be measured with the platelet aggregation rate and serum TLR4, MyD88, NF-*κ*B, COX-2, IL-6, CRP, TXB2 level for clinical efficacy and mechanisms at baseline and the 14^th^, 30^th^ day of treatment. (Fig. 1, Table 1).

**Table 1:** Schedule of enrollment, interventions and assessments.

Study procedure	Study period		
	Enrollment（d）	treatment period （d）	
Timepoints	-5^th^-0d	14^th^d ± 1d	30^th^d ± 1d
Enrolment			
Demographics	X		
Vital signs	X		
Medical history	X		
Complications	X		
Eligibility screen	X		
PHQ-9	X		
GAD-7	X		
MMSE	X		
Informed consent	X		
Random allocation	X		
Interventions			
Conventional treatments + Panax notoginseng		X	X
Conventional treatments		X	X
Assessments			
Platelet aggregation rate	X	X	X
TLR4, MyD88, NF-*κ*B, COX-2, IL-6, CRP, TXB2	X	X	X
Coagulation function	X	X	X
Safety evaluations	X	X	X
Patient compliance	X		
Drug combinations	X		
Adverse events	X		
Research summary	X		
Review statements	X		

GAD = generalized anxiety disorder, MMSE = mini-mental state examination, PHQ = patient health questionnaire.

The clinical trial results will be reported according to the Standards for Reporting Interventions in Clinical Trials of Chinese Herbal Medicine Formulae 2017 (CONSORT-CHM extension 2017) statement.^[11]^

### 2.2. Ethics

The study protocol (version: V1.0) has been approved by the ethics committee of *Xi’an Hospital of Traditional Chinese Medicine (Xi’an Affiliated Hospital of Shaanxi Provincial Hospital of Chinese Medicine*) (approval NO.: LLSCPJ2021008) at May 18, 2021, and registered on http://www.chictr.org.cn/index.aspx developed by the Chinese Clinical Trial Registry with the ID ChiCTR2100045773 at April 24, 2021. Informed consents will be obtained from all the subjects involved. Any modification of the study protocol or informed consent that may affect the rights and interests of the participants or the implementation of this trial shall be reported to the Ethics Committee for approval. In case of any serious adverse event (AE) in the trial, the ethics committee shall promptly review it and recommend written modifications, including sufficient authority to suspend the trial.

### 2.3. Study population

#### 2.3.1. *Inclusion criteria*.

1.Subjects, diagnosed with ischemic stroke, according to the *International Classification of Diseases (ICD-11, 8B11*),^[12]^ and *Chinese Guidelines for Diagnosis and Treatment of Acute Ischemic Stroke 2018*;^[13]^

2. Subjects, diagnosed with aspirin semi-resistance, according to 0.5 mmol/L arachidonic acid-induced mean platelet aggregation rate ≥ 20%, or 10*μ*mol/L adenosine diphosphate-induced mean platelet aggregation rate ≥ 70%;^[4,14]^

3. Subjects, aged between 45 and 65 years old, male or female;

4. Subjects, predicted in the low-risk profile of recurrent stroke, Essen stroke risk scores ≤ 3 points;^[15]^

5. Only taking single antiplatelet drug, not overlapping the use of activating blood and removing stasis herbs medicine;

6.Sign the informed consent forms.

#### 2.3.2. *Exclusion criteria*.

1.Subjects, diagnosed with transient ischemic attack or cerebral infarction who need to take 2 antiplatelet drugs;

2. Cerebral infarction combined with cerebral hemorrhage, cerebral arteritis, brain tumor, brain trauma, brain parasitic disease, and rheumatic heart disease, et cetera;

3. With bleeding tendency or severe bleeding symptoms within 3 months;

4. With severe kidney and liver function insufficiency & hematopoietic and metabolic diseases;

5. With disabilities, such as blindness, deafness, dumb, mental disorders (depression [patient health questionnaire-9 scores ≥ 20points], anxiety [generalized anxiety disorder-7 scores ≥ 19points], cognitive dysfunction [mini-mental state examination scores＜17 points])^[16,17]^ and non-stroke physical disabilities;

6. Abuse of alcohol and (or) drugs, or other conditions that affect compliance;

7. Participation in any other clinical trial in recent 3 months.

#### 2.3.3. *Withdrawal criteria*.

1.Violation of the inclusion criteria & clinical trial protocol;

2. Receive < 50% of treatment sessions;

3. Serious AEs or deterioration of stroke;

4. Withdraw the signed informed consent by subject or legal representative;

5. Replace the treatment protocol and (or) receive other unrelated treatments that could affect the study results.

### 2.4. *Study settings and recruitments*

The present study will be conducted in the Xi’an Hospital of Traditional Chinese Medicine (Xi’an Affiliated Hospital of Shaanxi Provincial Hospital of Chinese Medicine) and Shanxi Provincial Hospital of Chinese Medicine (Shaanxi Academy of Traditional Chinese Medicine).

Cerebral infarction patients with ASR in accord with the inclusion-exclusion criteria will be recruited at above-mentioned 2 hospitals, who mainly come from hospital wards & clinics. In addition, posters, leaflets and routine free clinics (every month) will be also helpful. Sure, the hospital websites, WeChat, and Microblogging are powerful advocacy media.

### 2.5. *Study group*

106 participants who meet above-mentioned criteria with signed informed consents will be selected. The subjects will be randomly divided into 2 groups in a 1:1 ratio, namely the experimental group and control group, 53 cases in each group.

### 2.6. *Study time*

This trial will be conducted from June 1, 2021 to May 31, 2023.

### 2.7. *Interventions*

Interventions are selected base on the theory of blood stasis in traditional Chinese medicine (TCM) and experience of experts.^[18]^ The traditional Chinese physicians involved have received their practitioners’ license from China’s National Health and Family Planning Commission, who have worked for more than 3 years. Subjects in experimental group will receive conventional treatments plus *Panax notoginseng (Sanqi*), and others in control group will just receive conventional treatments. Besides, if bleeding events or drug allergy happen during period, antiplatelet drug adjustment would be allowed.

#### 2.7.1. *Experimental group*.

##### 2.7.1.1. *Conventional treatments plus panax notoginseng (Sanqi*).

During the study period, patients will be given conventional treatments for ischemic stroke according to the *Guidelines for Secondary Prevention of Ischemic Stroke and transient ischemic attack in China (2014 edition*),^[19]^ such as taking an antiplatelet agent, anti-hypertensive drugs, lipid-lowering drugs, antidiabetic agents, intravascular treatments, folic acid and vitamins, et cetera. Nutritional support will be also allowed.

*Panax notoginseng (Sanqi)*, as a typical TCM, is recommended for the secondary prevention of cerebral infarction with blood stasis syndrome in the *Diagnosis and Treatment Guidance for Blood Stasis Syndrome by Integrative Medicine*^[18]^ and *Evidence-based practice guideline on integrative medicine for stroke 2019* (level of evidence: 2C).^[20]^ *Notoginseng* powder (云YPBZ-0091-2008, 3g, Yunnan Qiangfa Pharmaceutical Co. LTD, Kunming, China) will be administered after dissolved, 1 bag (3g) each time, once a day. Participants will be treated once a day for 30 consecutive days.

#### 2.7.2. *Control group*.

##### 2.7.2.1. *Conventional treatments*.

Patients in control group will be given conventional treatments mentioned above for ischemic stroke as same as the experimental group, once a day for 30 consecutive days.

### 2.8. *Adverse events observation*

It could be that patients with xerocheilia, irritability, bleeding symptoms, et cetera are more likely related to investigational medications. With the patients diary, all AEs are to be recorded and measured during treatment period. Serious AEs should be reported to the principal investigator (PI) immediately. All details will be documented, antiplatelet drug adjustment, as well as appropriate medical care will be given at once. For the subjects with severe stroke during study, they would be considered as drop outs.

### 2.9. Primary outcomes

The primary outcome will be the change in platelet aggregation rate of elbow venous blood for platelet adhesion properties at baseline, 14^th^ day and 30^th^ day of treatment; Moreover, the serum TLR4, MyD88, NF-*κ*B, COX-2, IL-6, CRP, TXB2 level for AR improvement based on the TLR4/MyD88/NF-*κ*B signaling pathway would be also detected at above-mentioned 3 time points.

### 2.10. Secondary outcomes

The secondary outcome will be the change of coagulation function for platelet assay and hemorrhage risk of stroke patients at 0 day, 14^th^ day and 30^th^ day of treatment. In addition, changes in liver and kidney function will be also tested for the medication safety in patients pre-and post-treatment. Their blood glucose, blood lipids, blood pressure, AEs and others will be tracked in patients diary during period. All the dropouts and causes will be documented in case report forms.

### 2.11. Estimation of sample size

The study is powered for the primary outcome measure of platelet aggregation rate. The number of patients required for the study was computed a priori with software PASS 15.0.1, NCSS, LLC, America. The sample size calculation was based on the results of preliminary small sample pilot trial by our team, which showed a decrease of-14% in platelet aggregation rate in the exercise group compared with control group (autocorrelation coefficient 0.7, 3 time points). A 1-sided 0.05 level of significance and a sample size of 96 participants (48 per group) provided 80% statistical power to demonstrate this difference in platelet aggregation rate. To accommodate for a 10% attrition rate, we will recruit a total of 106 participants (53 per group) (PASS 15.0.1, 2-way repeated measures Anova; allocation ration = 1).

### 2.12. Randomization and allocation concealment

After recruitment, patients who meet the eligibility criteria would be randomly and equally divided into 2 groups at a ratio of 1:1 with computer-based random sequence. Each subject will be registered with a unique ID. After screening and informed consent forms obtaining, subjects will be assigned to experimental group (conventional treatments plus *Panax notoginseng [Sanqi]*), control group (conventional treatments) randomly. Allocation was concealed in opaque envelopes until the beginning of intervention. Each subject will receive different medications, whereas all subjects will be aware of the group.

### 2.13. Statistical analysis

Data analysis will be based on the full analysis set and per-protocol set in the light of data missingness (last-observation-carried-forward [LOCF] method) and robustness of our analytical results. All data will be analyzed using IBM SPSS Statistics 21.0 (v21.0, IBM Corp., Armonk, NY). Descriptive statistics will be performed with means ± standard deviations or median [interquartile range]. Baseline characteristics of patients will be summarized by groups and compared with Chi-square for categorical variables, and Student’s independent *t* test or nonparametric Mann-Whitney U test for the continuous variables. Primary and secondary outcomes are applicable to 2-way repeated measures Anova, and Post Hoc test will be for multiple comparisons between groups and time-points. If there is a violation of distribution assumption, appropriate transformation will be used. A *P* value < 2- sided 0.05 will be considered as significant.

#### 2.13.1. *Primary outcomes*.

Platelet aggregation rate, serum TLR4, MyD88, NF-*κ*B, COX-2, IL-6, CRP, TXB2 level as primary outcomes, which are tested at baseline, 14^th^ and 30^th^ day of treatment, will be analyzed with 2-way repeated measures Anova. A significant effect of group indicates that the platelet aggregation rate & TLR4/MyD88/NF-*κ*B signaling pathway associated proteins are different between groups after intervention.

#### 2.13.2. *Secondary outcomes*.

Coagulation tested at above-mentioned 3 time points, will be also analyzed with 2-way repeated measures Anova.

#### 2.13.3. *Safety evaluation*.

The number (n) and percentage (%) of participants with AEs recorded in the diary will be calculated and compared using Chi-square test.

### 2.14. *Quality control*

Reasons of dropouts during research periods will be fully recorded for data analysis. To ensure quality, the PI (Hui Wang) and sub-PI (Jie Yuan) will verify all study process every 2 months and check data authenticity every 2 week on-site or online. Moreover, a third party, scientific research department of *Xi’an Hospital of Traditional Chinese Medicine (Xi’an Affiliated Hospital of Shaanxi Provincial Hospital of Chinese Medicine*), will be invited to manage the data independently.

Since this study mainly involves some objective indicators tested by device, measurement bias are mainly from different devices. In order to increase consistency, on the 1 hand, blood samples will be taken from patients at fixed time (7:30 am) by a fixed nurse. On the other hand, blood samples from the sub-center (*Shanxi Provincial Hospital of Chinese Medicine [Shaanxi Academy of Traditional Chinese Medicine]*) will be delivered to our hospital(*Xi’an Hospital of Traditional Chinese Medicine [Xi’an Affiliated Hospital of Shaanxi Provincial Hospital of Chinese Medicine]*) on the day for unified storage and detection. Moreover, record cards for all patients will be prepared for treatment history, including treatment date, personal information and their signatures.

## 3. Discussion

The occurrence of AR does increase the recurrence risk of cerebrovascular events. Due to the complexity of its pathogenesis, there is still no consensus on its treatment.

Therefore, we hope to obtain an appropriate partial substitute of aspirin for AR individuals through the advantages of multi-target and multi-pathway of TCM. From the perspective, anti-platelet aggregation drugs belong to the category of activating blood and removing stasis herbs medicine in TCM.^[21]^ So, activating blood and removing stasis herbs medicine may reduce the incidence and recurrence of stroke.

Herb medicine *sanqi* is the dried roots and rhizomes of Panax notoginseng. In clinic, it is mainly used for the treatment of diseases of cardio-cerebral system and vascular system.^[22]^ Modern pharmacological studies suggest that *Panax notoginseng (Sanqi*) can improve brain circulation and protect brain tissue. *Panax notoginseng (Sanqi*) powder can be used for cerebral infarction by the effect of activating blood and removing stasis.^[23]^ Its main active components, total saponins of *Panax notoginseng (Sanqi*), can effectively reduce blood lipid - glucose content, dilate blood vessels, decompose and inhibit the small thrombi in vessels with the enzyme- promoted cascade reaction, which has a certain effect on stroke.^[22]^

A great deal of experimental evidence indicates that inflammatory injury exerts a significant impact on the development of cerebral ischemia.^[24]^ During cerebral ischemia, TNF-*α*,IL-1,IL-6, et cetera, involved in inflammation, which would cause irreversible damage to brain. NF-*κ*B plays an important role in the inflammation. Phosphorylation of I*κ*B leads to phosphorylation of NF-*κ*B, which initiates transcription, regulates expression of cellular inflammatory factors, and promotes inflammation. In addition, inflammatory factors and adhesion molecules can induce further NF-*κ*B activation and aggravate the inflammatory process.^[25]^ Studies have shown that total saponins of *Panax notoginseng (Sanqi*) can inhibit the NF-*κ*B activation, block the I*κ*B phosphorylation, reduce the NF-*κ*B transcription into the nucleus, inhibit the expression of TNF-*α* in ischemic brain tissue.^[26]^ TLR4, a member of the signal transduction family, binded to the adaptor protein MyD88 and interleukin-related kinases to activate the TLR4/MyD88/NF-*κ*B signaling pathway could induce inflammatory responses.^[27]^ Study shows that it may ameliorate cerebral ischemia injury by decreasing expression of TLR4, MyD88 and NF-*κ*B P65 protein via inhibiting the TLR4/MyD88/NF-*κ*B P65 signaling pathway.^[28]^ Therefore, the possible mechanism of *Panax notoginseng (Sanqi*) for AR in stroke may be by inhibiting the inflammatory TLR4/MyD88/NF-*κ*B pathway.

Our study protocol exists several limits, such as open-blinding, short observation time, and without dose-effect relationship. However, we will try our best to attempt to minimize the biases that may influence study results. Finally we intend to demonstrate the hypothesis that *Panax notoginseng (Sanqi*) as an alternative may be an appropriate partial substitute of aspirin for AR individuals via TLR4/MyD88/NF-*κ*B signaling pathway.

## Author contributions

**Data collection:** Hui Wang, Jie Yuan,Ying Wang.

**Formal analysis:** Hui Wang,Ying Wang.

**Funding acquisition:** Jie Yuan, Jie Chen.

**Investigation:** Jie Yuan.

**Methodology:** Hui Wang, Jie Yuan.

**Project administration:** Hui Wang, Jie Yuan.

**Writing ‐ original draft:** Hui Wang and Jie Chen.

**Writing ‐ review & editing:** Hui Wang, Jie Chen.

## Supplementary Material
